# The complete plastid genome of *Kelloggia chinensis* Franch. (Rubiaceae), an endemic species from East Asia

**DOI:** 10.1080/23802359.2021.2009387

**Published:** 2021-12-20

**Authors:** Li-E Yang, Xiao-Juan Li, De-Li Peng

**Affiliations:** a Faculty of Geography, Yunnan Key Laboratory of Plateau Geographical Processes and Environmental Change, Yunnan Normal University, Kunming, China; b Key Laboratory of Plant Resources Conservation and Sustainable Utilization, South China Botanical Garden, Chinese Academy of Sciences, Guangzhou, China; c School of Life Sciences, Yunnan Normal University, Kunming, China

**Keywords:** *Kelloggia chinensis* Franch., endemic species, Rubiaceae, phylogeny, plastid genome

## Abstract

*Kelloggia chinensis* Franch. is an herbal plant species endemic to East Asia. Its complete plastid genome sequence is 155, 665 bp in length, with a large single-copy (LSC) region of 85, 788 bp, a small single-copy (SSC) region of 16, 977 bp, and a pair of inverted repeat regions (IRs) of 26, 450 bp. The whole plastid genome contains 132 genes, including 87 protein-coding genes, 37 tRNA genes, and 8 rRNA genes. The overall GC content of *K. chinensis* plastid genome is 37.1%. *K*. *chinesis i*s evolutionarily close to tribe Rubieae according to the Maximum likelihood phylogenetic analysis based on 12 taxa.

*Kelloggia* Torrey ex Bentham is a small genus of Rubiaceae. It consists of only two species: *Kelloggia chinensis* Franch. from East Asia (Dempster 1975) and *Kelloggia galioides* Torrey from the western United States (Bentham and Hooker 1873). This genus shows a disjunction between eastern Asia and western North America. *K*. *chinensis* is a perennial herb occurs in alpine meadows or forest clearances at above 3, 000 m in East Asia (Chen and Ehrendorfer 2011). In the present study, we reported the first complete plastid genome of *K. chinensis* and performed the phylogenetic analysis with other related species within the subfamily of Rubioideae based on the plastid genome sequences.

Fresh leaves were collected from Daochen county, Sichuan province of China (99°42′55.03″E, 29°02′47.64″N). A specimen was deposited at the Herbarium of Yunnan Normal University (YNUB, lieyang90@163.com) under the voucher number YangLE649. Total genomic DNA was extracted from leaf tissue with an improved 4 x CTAB method (Doyle and Doyle 1987). Illumina paired- end (PE) library was constructed, and high-throughput genome sequencing was performed on the Illumina HiSeq X Ten platform. The GetOrganelle v1.7.0 (Jin et al. 2020) and the PGA (Qu et al. 2019) were used to assemble and annotate the chloroplast genome, respectively, with the whole plastid genome sequences of *Galium mollugo* L. (KY562588) as reference. The newly annotated complete plastid genome was submitted to GenBank (accession number OK236359).

The complete plastid genome of *K. chinensis* is 155, 665 bp in length, containing a pair of inverted repeats (IRs) of 26, 450 bp, a large single-copy (LSC) region of 85,788bp and a small single-copy (SSC) region of 16, 977 bp. The overall GC content of this genome is 37.1% (LSC, 37.1%; SSC, 31.3%; IRs, 42.9%). The whole plastid genome contains 132 genes, including 87 protein-coding genes, 37 tRNA genes, and 8 rRNA genes. Among the annotated genes 16 genes (*atpF, ndhA, ndhB, rpl2, rpoC1, rps12, rps16, petB, petD, trnA-UGC, trnG-GCC, trnI-CAU, trnI-GAU, trnK-UUU, trnL-UAA, trnV-UAC*) comprise one intron, and two introns are contained in clpP and ycf3.

To clarify the phylogenetic position of *K. chinensis*, the complete plastid genomes of 10 species were selected within the subfamily of Rubioideae. Another two species from close subfamily were chosen as outgroups. The plastomes of the 12 accessions were aligned using MAFFT (Katoh and Standley 2013). A maximum likelihood analysis was performed using RAxML (Stamatakis 2006) software with GTR + G model and using the rapid bootstrap with 1000 replicates. The tribe of Rubieae is supported as a monophyly clade by the phylogenetic analysis, and *K. chinensis* is evolutionarily close to trirbe Rubieae and it is the sister group of this tribe (Figure 1). This report provided a valuable resource for the future studies in *Kelloggia* and related taxa.

**Figure 1.**
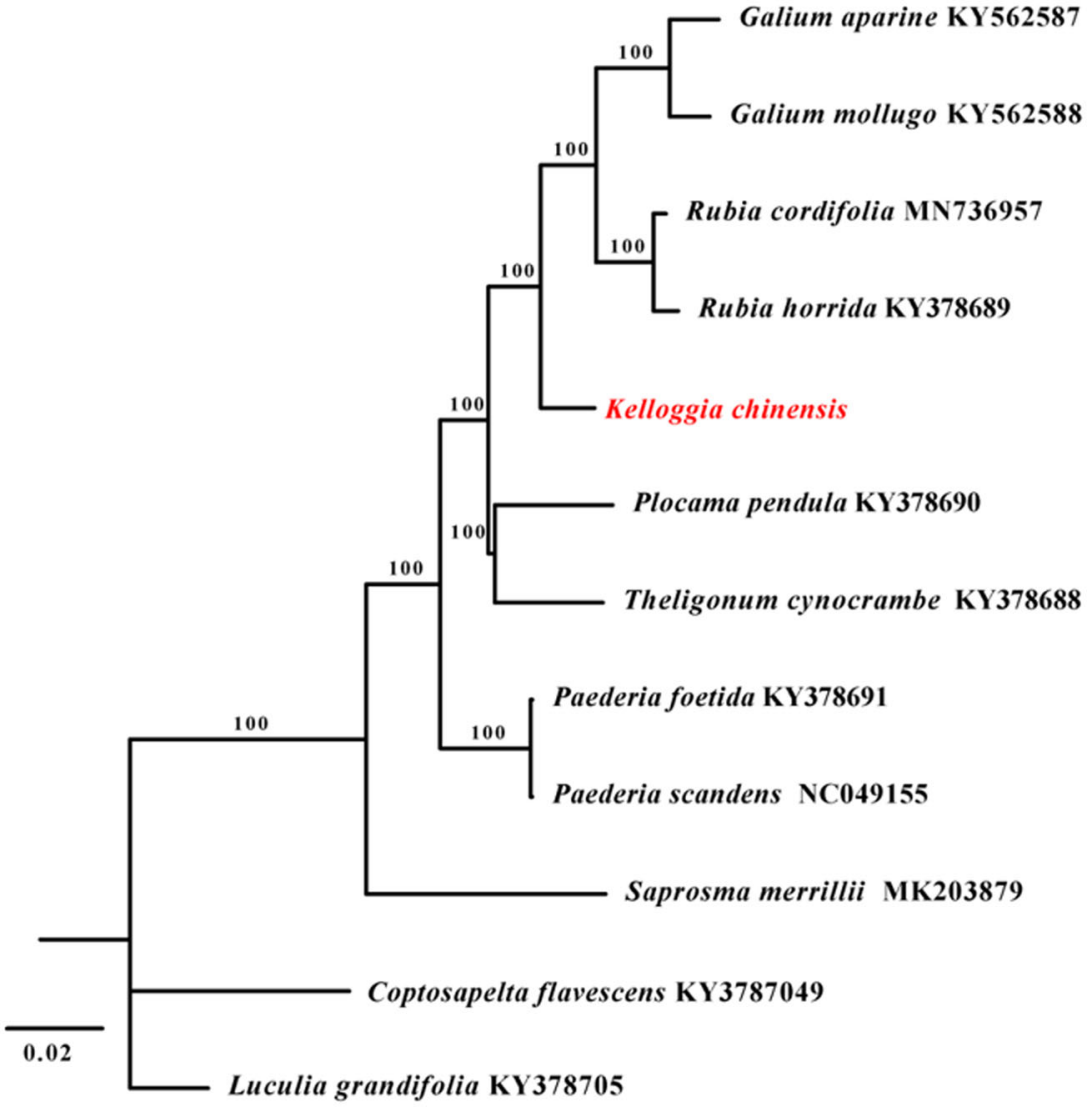
The maximum likelihood (ML) tree based on complete plastid genome sequences from 10 species within the subfamily of Rubioideae. *Luculia grandifolia* (KY378705) and *Coptosapelta flavescens* (KY3787049) were used as outgroups. The bootstrap support values with 1000 replicates are shown at each node.

## Data Availability

The genome sequence data that support the findings of this study are openly available in GenBank of NCBI at [https://www.ncbi.nlm.nih.gov] (https://www.ncbi.nlm.nih.gov/) under the accession no. OK236359. The associated BioProject, SRA, and Bio-Sample numbers are PRJNA776541, SRR16643418, and SAMN22794791 respectively.
